# Norman Francis Clifford Burgess

**Published:** 1940

**Authors:** 


### Obituary

### NORMAN FRANCIS CLIFFORD BURGESS, M.A., M.D., M.R.C.P., Lieutenant-Colonel R.A.M.C.(T.).

THE death of Norman Burgess on 18th October, 1940, at the early age of 38, has robbed Bristol and the neighbourhood of a distinguished Dermatologist, and his colleagues of a friend whom they held in deep affection. He was born in Bristol and educated at Clifton College, where in 1919 he gained an exhibition at Gonville and Caius College, Cambridge. There, like many other Caius men, he not only studied medicine but also took the keenest interest in the O.T.C. and raised the University Medical Unit from zero to sixty members. From that time onwards he never lost his zest for the Territorial branch of the R.A.M.C. This interest never led him to slacken his studies and he entered Guy's Hospital from Cambridge as Senior Science Scholar. Subsequently he became Poulton Research Student and Arthur Durham Travelling Student at Guy's, and in 1931 was awarded a B.M.A. research scholarship.

He qualified M.R.C.S. in 1924, took his M.A. together with the M.B., B.Ch. in 1926, and became M.R.C.P.(Lond.) in the same year. He proceeded to the M.D. in 1929. Whilst holding the Travelling Studentship Burgess worked in the University of Pennsylvania, Paris and Vienna.

In 1929 he was appointed Police Surgeon to the Bristol Constabulary and Assistant Physician in the Skin Department of the Bristol General Hospital. On the retirement of Dr. Kenneth Wills in 1932 he was made Physician-in-Charge of the Skin and Light Departments at the General Hospital. He was also Dermatologist at Southmead, Cossham, and the Bristol Homœopathic Hospitals.

The work of the St. John Ambulance Brigade won his wholehearted support and at the time of his death he was Commissioner for Bristol. The Order of St. John of Jerusalem was conferred on him in recognition of this work.

After serving as Regimental Medical Officer to the 4th Gloucesters (T.A.) for many years he was mobilised with that battalion at the outbreak of the war. In June, 1939, he was promoted Senior M.O. to an A.A. Brigade with the rank of lieutenant-colonel.

His published writings were numerous and were typical of his industry and his modern scientific outlook on dermatology.

A man of undoubted push and go Norman Burgess pursued a successful career without inspiring any feelings of envy and in singular friendliness with his own profession. His masterfulness was enhanced by a winning manner and a happy temper. He bore everyone, even fools, gladly, a tribute that cannot always be paid to those who achieve success.

He married, in 1930, Helen Harris, of Hamilton, Ontario, who with one son and two daughters survives him. To his widow and children, and also to his parents, we offer our deepest sympathy.

